# Bioinformatic analysis confirms differences in circular RNA expression profiles of cumulus cells between patients with ovarian and peritoneal endometriosis-associated infertility

**DOI:** 10.3389/fendo.2023.1137235

**Published:** 2023-03-15

**Authors:** Xiaodi Huang, Qi Yu

**Affiliations:** Department of Obstetrics and Gynecology, Peking Union Medical College Hospital, Chinese Academy of Medical Sciences and Peking Union Medical College, National Clinical Research Center for Obstetric and Gynecologic Diseases, Beijing, China

**Keywords:** circular RNAs, ovarian endometriosis, peritoneal endometriosis, expression profiling, competitive endogenous RNA, cumulus cells

## Abstract

Endometriosis has a detrimental effect on oocyte quality, and ovarian endometriosis (OEM) and peritoneal endometriosis (PEM) may have different effects on female fertility. Therefore, we conducted a study to explore the circular RNA (circRNA) expression profiles of cumulus cells (CCs) in patients with OEM (n = 3), PEM (n = 3), and tubal factor infertility (TFI, n = 3) using high-throughput sequencing techniques and attempted to identify common and unique circRNAs in the OEM and PEM groups. The CIRCexplorer2 program was used to identify circRNAs. Seven candidate circRNAs were validated in 30 samples using quantitative real-time polymerase chain reaction (qRT-PCR). Finally, Gene Ontology (GO) and Kyoto Encyclopedia of Genes and Genomes (KEGG) analyses were performed to annotate the function of circRNA-targeted genes, which were verified by sequencing results and constructed circRNA–miRNA–mRNA networks. A total of 11833 circRNAs were identified in nine samples. The numbers of differentially expressed circRNAs between the OEM and TFI groups, PEM and TFI groups, and OEM and PEM groups were 130, 71, and 191, respectively. After taking intersections, 11 circRNAs were considered common circRNAs in the OEM and PEM groups; 39 circRNAs in the OEM group and 17 circRNAs in the PEM group were identified as unique key circRNAs. During qRT-PCR validation, hsa_circ_0003638 was significantly upregulated in the PEM group compared to that in the OEM and TFI groups. Functional analysis of circRNA-targeted genes revealed that apoptosis, PI3K-AKT, and p53 signaling pathways were enriched in the PEM–TFI comparison groups, whereas the functions of target genes involved in the JAK–STAT and TGF-β signaling pathways were enriched in the PEM–OEM comparison groups. Our findings confirmed differences in circRNA expression profiles of CCs between patients with OEM and PEM infertility and provide new insights into the different effects of various endometriosis phenotypes on oocytes.

## 1 Introduction

Endometriosis is closely related to infertility (1), and many patients seek assisted reproductive technology (ART); however, the pregnancy outcomes of patients with endometriosis remain adverse (2). Patients with endometriosis tend to have poor quality oocytes and embryos (3, 4), causing low pregnancy rates. Thus, investigating the factors that influence oocyte quality in patients with infertility and endometriosis is critical. Cumulus cells (CCs) are somatic cells that surround oocytes and originate from undifferentiated granulosa cells (5). As a component of the cumulus–oocyte complex, CCs communicate constantly with the oocyte and influence its developmental competence (6). Therefore, gene expression profile analysis in human oocyte CCs can be used as a non-invasive approach for oocyte quality assessment (7).

Circular RNAs (circRNAs), a subset of long non-coding RNAs (lncRNAs), are characterized by a covalently closed loop structure without a 5’-3’ polarity or a poly(A) tail, which increases stability and resistance to RNase R (8, 9). In recent years, next-generation sequencing technology has accelerated the discovery of circRNAs, allowing investigation into their biological roles in gene regulation; for instance, circRNAs act as microRNA (miRNA) sponges to bind miRNAs in competitive endogenous RNA (ceRNA) networks (10, 11). Emerging evidence suggests that circRNAs may contribute to disease development, including endometriosis (8, 11). However, there are few studies on the function of circRNAs in CCs of patients with infertility along with endometriosis. To the best of our knowledge, there are no studies regarding the expression profiles of CCs in different phenotypes of endometriosis. Previous studies report that ovarian endometriosis (OEM) and peritoneal endometriosis (PEM) have different effects on female fertility; for instance, PEM is a risk factor for endometriosis-associated infertility while OEM is not, and OEM rarely induces inflammation in nearby follicles during *in-vitro* fertilization (12, 13). Hence, it is necessary to explore whether there are differences in circRNA expression profiles and functions between the two phenotypes.

In this study, we investigated circRNA expression profiles of CCs in patients with two different phenotypes of endometriosis (OEM and PEM) and tubal factor infertility (TFI) using high-throughput sequencing techniques. Subsequently, we constructed circRNA–miRNA–mRNA ceRNA networks and analyzed the function of circRNA-targeted genes using bioinformatic analysis. Our findings provide new evidence regarding the various effects of different endometriosis phenotypes on oocytes.

## 2 Materials and methods

### 2.1 Patient recruitment

This study was approved by the Institutional Review Board of the Peking Union Medical College Hospital (number: JS-3376). Informed consent was obtained in accordance with the institutional guidelines.

A total of 39 patients with infertility were enrolled from December 2021 to September 2022 at our hospital and divided into three groups of 13 patients each. In the OEM and PEM groups, endometriosis was surgically confirmed to ensure that the patients in each group had only one phenotype of endometriosis and not a mixture of the phenotypes. Endometriosis was classified according to the revised American Society for Reproductive Medicine criteria (14), and all lesions were removed prior to ART. In the OEM group, nine patients had stage III disease and four patients had stage IV disease, while in the PEM group, all patients had stage I disease. All patients in the control group had TFI without endometriosis lesions, as confirmed by surgery.

Other inclusion criteria included: age between 20 and 40 years, normal ovulation, baseline follicle stimulating hormone (FSH) levels< 10 IU/L and anti-Müllerian hormone > 1.1 ng/mL; no hormone therapy within three months prior to ART, and body mass index (BMI)< 30 kg/m^2^. The exclusion criteria included ovulatory dysfunction; untreated hydrosalpinx; cardiovascular diseases; dyslipidemia; endocrine diseases, including thyroid disorder, hyperprolactinemia or diabetes mellitus; autoimmune diseases; history of tuberculosis; smoking, drug, and/or alcohol habits; and patients with ovarian malignant tumors (15–17).

### 2.2 Controlled ovarian stimulation protocols

The patients underwent controlled ovarian stimulation based on their age, BMI, ovarian reserve, and previous ovarian response. Most patients in the TFI and PEM groups underwent gonadotropin-releasing hormone (GnRH) antagonist protocols, whereas most patients in the OEM group underwent long GnRH agonist protocols. Gonadotropins utilized in this study included recombinant FSH (Gonal-F, Merck Serono, Geneva, Switzerland), recombinant luteinizing hormone (Luveris, Merck Serono), and human menopausal gonadotropin (Livzon, Guangdong, China). Recombinant human chorionic gonadotropin (Ovidrel, Merck Serono) was used to trigger ovulation, when transvaginal ultrasonography detected the presence of at least three follicles larger than 16 mm. Subsequently, oocyte retrieval was performed 36 h later in the outpatient operating room.

### 2.3 Cumulus cell collection and purification

On the day of oocyte retrieval, CCs were collected and purified, as previously described (16, 18). Briefly, following follicular puncture, cumulus–oocyte complexes were collected in G-MOPS medium (Vitrolife, Goteborg, Sweden) under a microscope by experienced ART laboratory personnel. After mechanically stripping the oocytes under microscope, the remaining CCs were immediately transferred to a 15-mL disposable sterile centrifuge tube containing phosphate-buffered saline, and centrifuged at 500 × *g* for 2 min. After centrifugation and removal of the supernatant, 3–5 times the cell volume of red blood cell lysis buffer (Beyotime, Shanghai, China) was added to the pellets. The tube was maintained at 23–26°C, occasionally agitated for 1–2 min and centrifuged at 500 × *g* for 5 min. CCs were then washed twice with phosphate-buffered saline and transferred to a 1.5-mL Eppendorf tube. TRIzol reagent (Invitrogen, Carlsbad, CA, USA) was immediately added to the EP tube to prevent RNA degradation, and the samples were stored at −80°C for subsequent RNA extraction.

### 2.4 Total RNA extraction, library construction, and sequencing

Library construction and Illumina sequencing were performed by the Shanghai Lifegenes Technology Co., Ltd. (Shanghai, China). Briefly, total RNAs were extracted using the TRIzol reagent kit, following the manufacturer’s protocol. The purity and concentration of RNA were assessed using a NanoPhotometer spectrophotometer (IMPLEN, Munich, Germany). Moreover, the integrity was measured using the Agilent fragment analyzer 5400 system (Agilent Technologies, Santa Clara, CA, USA). A total amount of 3 μg RNA per sample was used for the strand-specific library construction. First, ribosomal RNA (rRNA) was removed using the Epicentre Ribo-zero rRNA removal kit (Epicentre, Madison, WI, USA), according to the manufacturer’s instructions. Subsequently, the sequencing library was generated from the rRNA-depleted RNA using the NEBNext ultra directional RNA library prep kit for Illumina (NEB, Ipswich, MA, USA) according to the manufacturer’s instructions. When synthesizing the second strand of cDNA, dTTP was replaced by dUTP and the USER Enzyme (NEB) was then used to degrade the second strand of cDNA containing the U base, which allowed us to determine whether the transcript was from the positive-sense or antisense DNA strand. Subsequently, the products were purified using the AMPure XP system (Beckman Coulter, Brea, CA, USA), and the quality of the library was assessed using the Agilent fragment analyzer 5400 system (Agilent Technologies). The qualified library was sequenced on an Illumina NovaSeq 6000 platform following the manufacturer’s instructions and 150 bp paired-end reads were generated. Finally, we obtained raw reads, including the sequences of circRNAs, mRNAs and lncRNAs. We did not obtain the miRNAs sequences because the RNA content in each sample was not sufficient to construct another sequencing library.

### 2.5 Data processing and circRNA and mRNA data analyses

The obtained raw reads of Fastq format were processed using in-house PERL scripts. In this step, we obtained high-quality clean reads for further analysis by removing reads containing adapters, poly-N, and low-quality reads. HISAT2 (version 2.2.1) was used for mapping the clean reads to the reference genome with parameter “rna-strandness RF” (19). The reference genome used for alignment was the GRCh38 version from the Ensembl database.

circRNAs were identified using the CIRCexplorer2 program (version 2.3.8) with default parameters (20). The circRNAs were then searched in circBase for annotation, and those that could not be found were identified as novel circRNAs named by their source genes (21). Since circBase uses the hg19 version of the genome, CrossMap was used to transform the aligned circRNA genome coordinates from GRCh38 to hg19 version. The expression levels of circRNAs in each sample were estimated by transcript per million value using the following equation (22):


(1) Normalized expression = mapped read count/total reads × 1000000

Subsequently, the limma R package (version 3.46.0) was used for differential expression analysis between the two groups. circRNAs with fold change (FC) ≥ 2 and P< 0.05 were considered as statistically differentially expressed circRNAs (DECs).

In addition, differential expression analysis of the mRNAs was performed for subsequent ceRNA network construction. After calculating the expected number of fragments per kilobase of transcript sequence per million base pairs sequenced (FPKM) value in each sample using StringTie (version 2.1.6) (23), the DESeq2 R package (version 1.32.0) was used for differential expression analysis between each of the two groups. mRNAs with FC ≥ 2, P< 0.01 and FPKM ≥ 1 in at least one sample were considered as significantly differentially expressed mRNAs.

### 2.6 Finding common and respective unique circRNAs in the OEM and PEM groups

To explore the impact of different phenotypes of endometriosis on oocytes, we attempted to identify common and unique circRNAs in the OEM and PEM groups. After taking the intersection of the three clusters of DECs, the circRNAs that were shared by the OEM and PEM groups with consistent up/down-regulation were considered common circRNAs that might contribute to oocyte damage in patients with OEM and PEM. Meanwhile, the circRNAs that were significantly upregulated or downregulated in the OEM group compared with the other two groups were selected, as they might be involved in the unique effect on oocytes in patients with OEM. The unique circRNAs in the PEM group were screened in the same manner.

### 2.7 Quantitative real−time polymerase chain reaction validation

During quantitative real-time polymerase chain reaction (qRT-PCR) validation, we selected circRNAs for validation based on the following aspects: the FC and P values of circRNAs; the source genes of circRNAs (24); the length of circRNAs; and previous reports about the circRNAs. Finally, two circRNAs (hsa_circ_0003638 and hsa_circ_0005205) from the common circRNA assemblage of the OEM and PEM groups, three circRNAs (hsa_circ_0005015, hsa_circ_0138839, and hsa_circ_0003221) from the OEM unique assemblage, and two circRNAs (hsa_circ_0004872 and hsa_circ_0008927) from the unique assemblage of PEM were chose for validation. qRT-PCR was performed in 10 samples in each group.

Total RNA was isolated from the samples that had been treated with TRIzol reagent and then reverse transcribed into cDNA using Superscript III reverse transcriptase (Invitrogen). The primers of the seven candidate circRNAs were synthesized by BGI BEIJING Co. (Beijing, China) (**Supplementary Table 1**), and β-actin was used as a reference gene. The design of the primers was in the light of the back-spliced junction sites of circRNAs. qRT-PCR was performed using 2 × PCR mix (QIAGEN, Venlo, Netherlands) in an ABI ViiA 7 RT-PCR system (Applied Biosystems, Carlsbad, CA, USA) according to the manufacturer’s instructions. Samples with more than 30 cycle threshold (CT) values were discarded to prevent false positives and make the results more convincing. The relative circRNA expression levels were calculated using the 2^-ΔΔCt^ method (25). The relative expression levels of the selected circRNAs are presented as the mean ± standard error of the mean. Student’s *t*-test was performed if the data were normally distributed; otherwise, the Mann–Whitney U test was used for analysis. Statistical significance was set at P< 0.05. Statistical analysis was performed using the SPSS version 23.0 software (IBM, Armonk, NY, USA), and the results were displayed using GraphPad Prism version 9.4.0 (GraphPad Software, San Diego, CA, USA).

### 2.8 Construction of ceRNA networks

When constructing the interaction network between circRNAs and miRNAs, miRanda (version 3.3a) was used to predict the miRNAs that could bind to the verified circRNAs, with the parameter (-en -30). We used two prediction algorithms, TargetScan (version 8.0) and miRDB, to predict the mRNAs that could bind the same miRNAs. Subsequently, the overlapped mRNAs that were significantly differentially expressed according to the sequencing results were selected. The selected mRNAs also needed to follow the principle of positive correlation with circRNA expression levels. Finally, the verified circRNAs, predicted miRNAs, and overlapped mRNAs were used to construct ceRNA networks. The ceRNA networks were visualized using Cytoscape software (version 3.7.1).

### 2.9 Gene Ontology and Kyoto Encyclopedia of Genes and Genomes enrichment analysis of circRNA-targeted genes

Based on the mRNAs in the ceRNA networks, Gene Ontology (GO) and Kyoto Encyclopedia of Genes and Genomes (KEGG) enrichment analyses were performed to annotate the functions and pathways of circRNA-targeted genes, respectively. GO analysis was performed using the clusterProfiler R package (version 3.12.0), and KOBAS software (version 3.0) was used to test the statistical enrichment in KEGG pathways (http://www.genome.jp/kegg/) (26). Statistical significance regarding enrichment in the GO and KEGG analyses was set as P< 0.05. Only the most significant GO terms that contained the same genes were retained. The top 15 significantly enriched biological process (BP) terms in GO analysis and pathways in KEGG analysis were determined.

## 3 Results

### 3.1 DECs in CCs from the three groups

**Figure 1:**
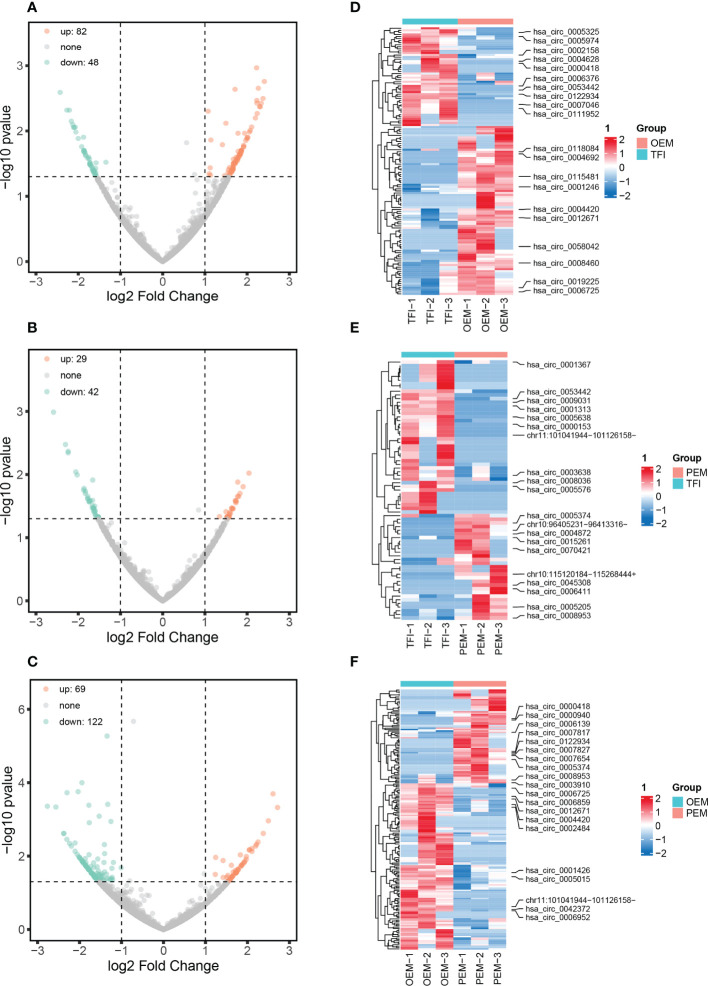
circRNA expression patterns of cumulus cells in patients with OEM (n = 3), PEM (n = 3), and TFI (n = 3). **(A–C)** Volcano plots showing all differentially expressed circRNAs between each of the two groups: **(A)** OEM and TFI; **(B)** PEM and TFI; and **(C)** OEM and PEM groups. **(D–F)** Heatmaps demonstrate the top 10 up- and downregulated circRNAs between the respective two groups. The positions of novel circRNAs are based on the hg 38 version. circRNAs, circular RNAs; OEM, ovarian endometriosis; PEM, peritoneal endometriosis; TFI, tubal factor infertility.

A total of 11833 circRNAs were identified from the nine samples, of which 3141 circRNAs were novel. The length of circRNAs was concentrated in the interval from 401 to 600 bp (**Supplementary Figure 1**). According to our results, 130 circRNAs were differentially expressed between the OEM and TFI groups, of which 82 were upregulated and 48 were downregulated in the OEM group. In the PEM group, 71 circRNAs (29 upregulated and 42 downregulated) were differentially expressed compared to those in the TFI group. A total of 191 circRNAs were found to be differentially expressed between the OEM and PEM groups, of which 69 were upregulated and 122 were downregulated in the PEM group. Volcano plots and heatmaps showed differences in circRNA expression levels between each of the two groups (**Figure 1**).

### 3.2 Finding common and respective unique circRNAs in the OEM and PEM groups

**Figure 2:**
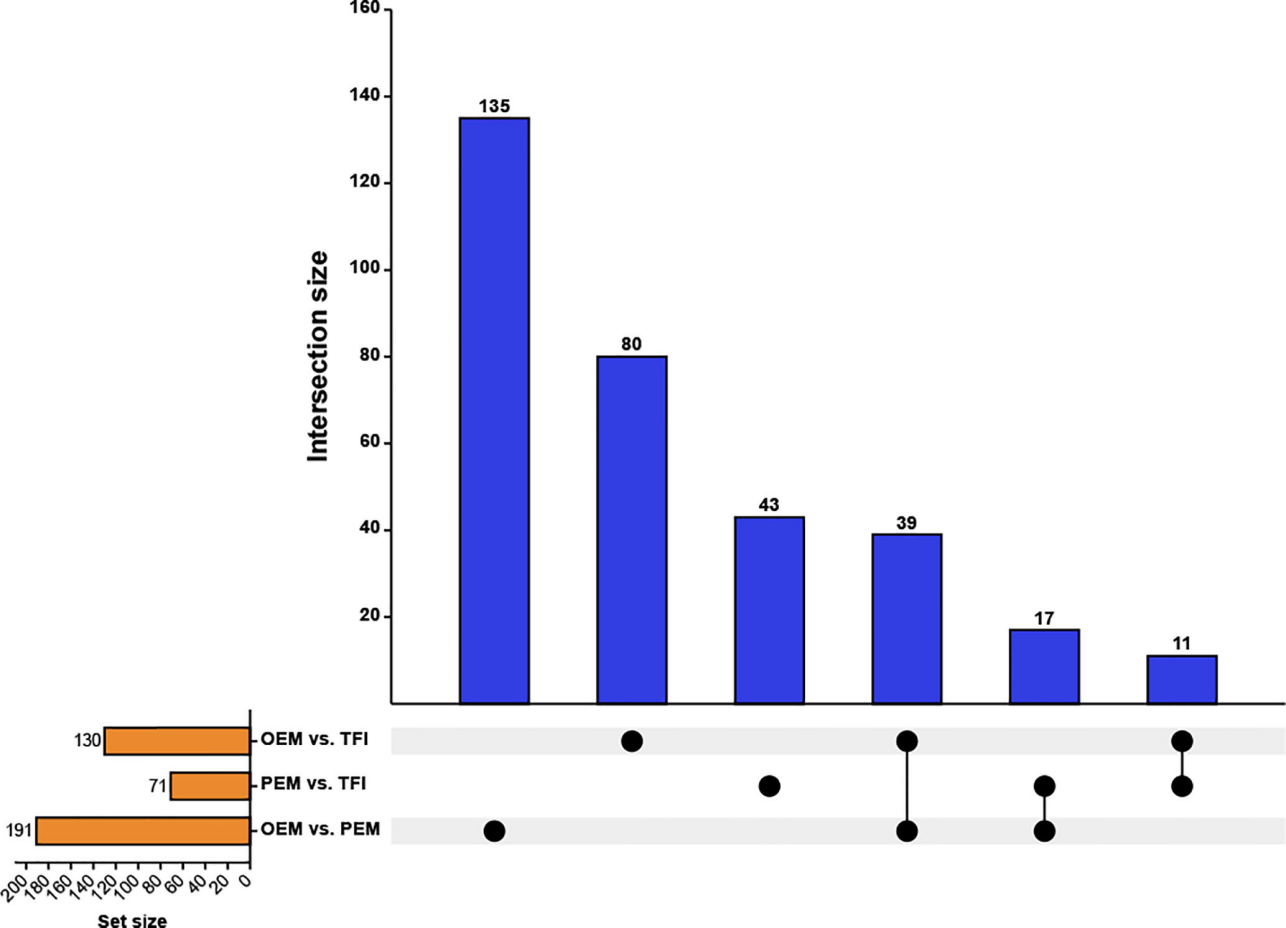
Upset plot depicting the intersections of the DECs. The blue columns represent the intersection size, and the yellow columns represent the size of the DECs of each comparison group. DECs, differentially expressed circular RNAs; OEM, ovarian endometriosis; PEM, peritoneal endometriosis; TFI, tubal factor infertility.

After taking the intersection of the DECs in each of the two groups (**Figure 2**), we found that 11 circRNAs were differentially expressed in the OEM and PEM groups compared to those in the TFI group, and the tendency of up/down-regulation was consistent. A total of 39 circRNAs were considered unique DECs in the OEM group, which were consistently upregulated or downregulated compared with that in the PEM and TFI groups. Similarly, 17 circRNAs were identified as unique differentially expressed circRNAs in the PEM group. **Supplementary Table 2** presents information on all circRNAs in the three assemblages.

### 3.3 qRT-PCR validation of selected circRNAs

**Figure 3:**
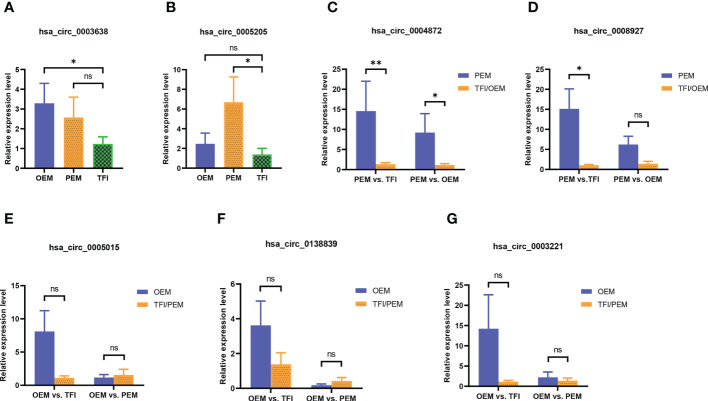
**(A–G)** The relative candidate circRNA expression levels of cumulus cells in patients with OEM (n = 10), PEM (n = 10), and TFI (n = 10) by qRT-PCR. **P < 0.01; *P < 0.05; ns, not significant. circRNAs, circular RNAs; OEM, ovarian endometriosis; PEM, peritoneal endometriosis; TFI, tubal factor infertility; qRT-PCR, quantitative real−time polymerase chain reaction.

We selected seven circRNAs for qRT-PCR validation in 30 samples; **Table 1** contains corresponding detailed information. According to our results (**Figure 3**), only hsa_circ_0004872 from the PEM unique cluster was significantly upregulated compared to the OEM and TFI groups, with P values of 0.038 and 0.005, respectively. hsa_circ_0003638 was found to be significantly upregulated in the OEM group compared to that in the TFI group (P = 0.035), which was opposite to the results of RNA sequencing; no difference was observed in the expression levels of hsa_circ_0003638 between the PEM and TFI groups (P = 0.412). The expression level of hsa_circ_0005205 was increased in the PEM group (P = 0.016) but was comparable in the OEM group (P = 0.413) compared with the TFI group. Another circRNA from the PEM unique cluster, hsa_circ_0008927, was marginally upregulated when compared with the TFI group (P = 0.047), but no significant difference in expression was found when compared with the OEM group (P = 0.132).

**Table 1:** Detailed information regarding the selected circRNAs for qRT-PCR validation.

circRNA name	circRNA position* (+ positive-sense strand/ - antisense strand)	circRNA source gene	Length (bp)	Regulation	Comparison group	FC	P value
has_circ_0003638	chr17:28163542-28172618+	*NLK*	398	down	OEM vs. TFI	0.27	0.020
					PEM vs. TFI	0.27	0.018
hsa_circ_0005205	chr14:61720378-61721823+	*HIF1A*	425	up	OEM vs. TFI	4.00	0.013
					PEM vs. TFI	3.56	0.020
hsa_circ_0005015	chr8:121628713-121629340-	*HAS2*	627	up	OEM vs. TFI	2.21	0.014
					OEM vs. PEM	2.51	0.000
hsa_circ_0138839	chr9:5064882-5081861+	*JAK2*	1515	up	OEM vs. TFI	4.00	0.013
					OEM vs. PEM	4.00	0.011
hsa_circ_0003221	chr8:140846259-140890769-	*PTK2*	625	up	OEM vs. TFI	2.67	0.016
					OEM vs. PEM	2.65	0.005
hsa_circ_0004872	chr22:21799011-21807846-	*MAPK1*	490	up	PEM vs. TFI	3.35	0.027
					PEM vs. OEM	3.35	0.027
hsa_circ_0008927	chr3:11358417-11380052+	*ATG7*	672	up	PEM vs. TFI	2.88	0.047
					PEM vs. OEM	2.88	0.050

*The positions of circRNAs were based on the hg 38 version. circRNAs, circular RNAs; qRT-PCR, quantitative real-time polymerase chain reaction; FC, fold change; OEM, ovarian endometriosis; PEM, peritoneal endometriosis; TFI, tubal factor infertility.

### 3.4 Construction of ceRNA networks

**Figure 4:**
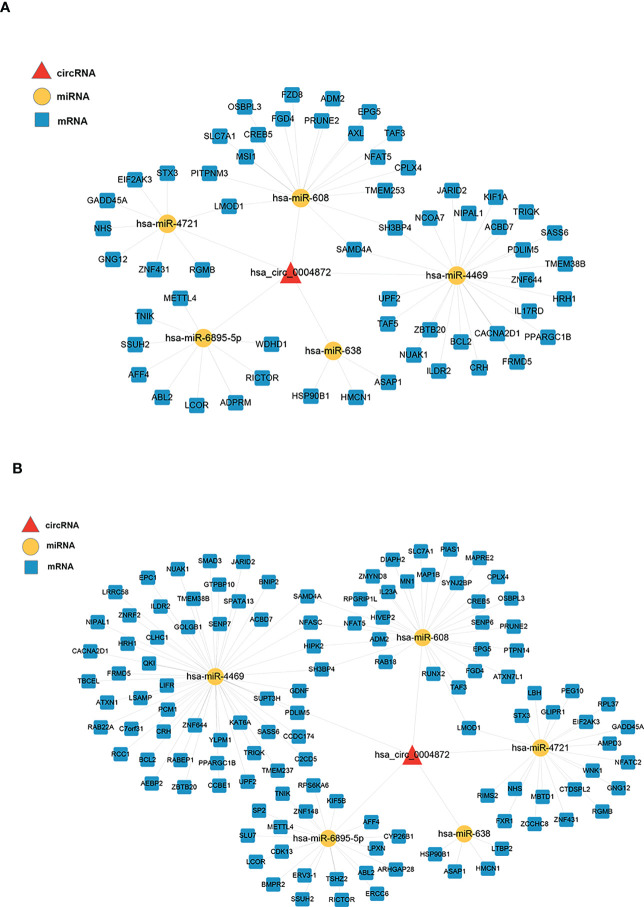
ceRNA networks constructed using the validated circRNAs, predicted miRNAs, and overlapped mRNAs. The circRNAs, miRNAs, and mRNAs are denoted by red triangles, yellow nodes, and blue round rectangles, respectively. **(A)** The PEM–TFI comparison groups and **(B)** PEM–OEM comparison groups. ceRNA, competitive endogenous RNA; circRNAs, circular RNAs; PEM, peritoneal endometriosis; TFI, tubal factor infertility; OEM, ovarian endometriosis.

We constructed two ceRNA networks based on hsa_circ_0004872, which were validated using qRT-PCR (**Figure 4**). Five miRNAs were predicted to bind to hsa_circ_0004872, namely hsa-miR-608, hsa-miR-638, hsa-miR-4469, hsa-miR-4721, and hsa-miR-6895-5p. The ceRNA network constructed by the PEM–TFI comparison groups contained 59 mRNAs, and 120 mRNAs were included in the network constructed by the PEM–OEM comparison groups.

### 3.5 GO and KEGG enrichment analysis of circRNA-targeted genes

**Figure 5:**
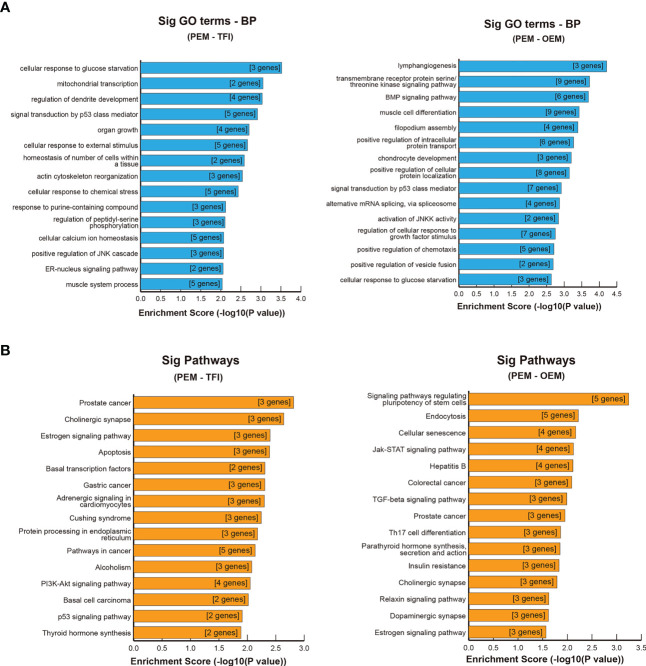
GO and KEGG enrichment analysis of circRNA-targeted genes. **(A)** Significant BP terms of GO analysis in the PEM–TFI and PEM–OEM comparison groups. **(B)** Significant KEGG pathways in the PEM–TFI and PEM–OEM comparison groups. The top 15 BP terms and pathways are displayed. GO, Gene Ontology; KEGG, Kyoto Encyclopedia of Genes and Genomes; circRNAs, circular RNAs; BP, biological process; PEM, peritoneal endometriosis; TFI, tubal factor infertility; OEM, ovarian endometriosis.

GO and KEGG enrichment analyses were performed for further functional investigation of the circRNA-targeted genes. In the PEM–TFI comparison groups, the results of GO analysis showed that these target genes were mainly involved in cellular response to glucose starvation, mitochondrial transcription, and signal transduction by p53 class mediators (**Figure 5A**). As for the PEM–OEM comparison groups, the mainly enriched BP terms included transmembrane receptor protein serine/threonine kinase signaling pathway, BMP signaling pathway, and positive regulation of intracellular protein transport (**Figure 5A**). According to the results of KEGG analysis, apoptosis and PI3K–AKT and p53 signaling pathways were significantly enriched in the PEM–TFI comparison groups (**Figure 5B**), whereas the JAK–STAT signaling pathway and TGF-β signaling pathway were included in the PEM–OEM comparison groups (**Figure 5B**).

## 4 Discussion

Endometriosis is a common benign disease usually accompanied by infertility problems in women of childbearing age. Endometriosis damages the quality of oocytes, leading to lower rate of mature oocytes, higher rates of morphologically abnormal oocytes and abnormal mitochondria (27–29). Thus, it is necessary to study the mechanisms underlying endometriosis-damaging oocytes. However, owing to the precision of human oocytes, direct use of oocytes for further studies is usually not feasible. CCs and follicular fluid provide an important intrafollicular environment that supports oocyte development (6). Intercellular dialogs between CCs and oocytes are accomplished by gap junctions and paracrine signals, and these contacts are critical for follicular growth, oocyte maturation, and oocyte competence acquisition (6). Because of this close relationship, CCs are often used as an indirect approach for studying oocytes.

Traditionally, endometriosis lesions have three phenotypes: peritoneal, ovarian (endometrioma), and deep (30). Different phenotypes may have varying effects on female fertility. A large observational, cross-sectional study including 2208 patients illustrated that peritoneal superficial endometriosis was a risk factor for endometriosis-associated infertility; however, endometrioma was not found to be a significant risk factor for infertility (12). Another retrospective study observed lower mature oocyte and fertilization rates in patients with PEM than in endometrioma-affected patients, confirming the different impacts of PEM and OEM on the acquisition of oocyte competence (31). Against the background of elevated levels of various proinflammatory cytokines in peritoneal fluid in patients with PEM, Opøien et al. evaluated the relationship between OEM and local intrafollicular inflammation by testing the cytokine concentrations in the follicular fluid (13). They found that cytokine levels in the follicular fluid were comparable among patients with unilateral endometriomas, bilateral endometriomas, and without endometriosis, indicating that OEM, whether unilateral or bilateral, does not induce an inflammatory environment in nearby follicles. However, there is a lack of studies on this issue; therefore, more evidence is needed to explore the different effects of PEM and OEM on female fertility, oocyte competence, and oocyte quality.

circRNAs, as members of non-coding RNAs, have been reported to regulate gene expression in different ways, such as binding miRNAs like sponges, regulating transcription, influencing splicing, and modifying the cellular function of bound factors as protein decoys (10). In recent years, some studies have shown that the expression profile of circRNAs in CCs is altered in patients with gynecological diseases. For instance, Ma et al. identified 286 DECs in CCs between patients with and without polycystic ovary syndrome, and three circRNAs (hsa_circ_0043533, hsa_circ_0043532, and hsa_circ_0097636) were validated to be differentially expressed by qRT-PCR (32). circRNA_103827 and circRNA_104816 were upregulated in CCs in women over 38 years old, and their expression levels were negatively correlated with embryo quality (33). Two studies performed by Wu et al. and Guo et al. confirmed the differences in circRNA expression profiles in CCs between patients with and without OEM (16, 34). Nonetheless, there are no studies on the circRNA expression profiles of CCs in patients with PEM, especially comparing the differences in circRNA expression profiles between patients with PEM and OEM infertility.

In this study, we explored the circRNA expression profiles of CCs in patients with infertility related to OEM, PEM, or TFI *via* high-throughput sequencing and compared the differences in circRNA expression between the (1) OEM and TFI groups, (2) PEM and TFI groups, and (3) OEM and PEM groups. The results showed that abundant circRNAs were identified, and approximately 26 percent of them were novel. We detected 130, 71, and 191 significantly DECs in the OEM–TFI, PEM–TFI, and OEM–PEM comparison groups, respectively, indicating that the circRNA expression profiles differed among the three groups. To investigate the similarities and differences in the effects of different endometriosis phenotypes on oocytes, we examined the intersections of DECs with consistent expression tendencies in each of the two comparison groups. Finally, we found 11 circRNAs involved in the common mechanisms of oocyte impairment in patients with OEM or PEM; 39 and 17 circRNAs were identified as unique key circRNAs in the OEM and PEM groups, respectively. Subsequently, seven circRNAs from three assemblages were selected for qRT-PCR validation. hsa_circ_0004872 was found to be significantly upregulated in the PEM group compared to both the OEM and TFI groups, implying that it might play a critical role in influencing oocyte quality and competence in patients with PEM. hsa_circ_0004872, spliced from the mitogen-activated protein kinase 1 (*MAPK1*), is downregulated in gastric cancer tissues and inhibits cancer cell proliferation and invasion by binding miR-224 or coding protein MAPK1-109aa (35, 36).

To date, the most studied function of circRNAs is to act as “sponges” to bind miRNAs and affect the expression levels of downstream target mRNAs, that is, to form ceRNA networks to regulate gene expression. Thus, we constructed ceRNA networks of hsa_circ_0004872 to identify sponged miRNAs and their downstream target genes. In the ceRNA network, five miRNAs were predicted to bind to hsa_circ_0004872. Among these, hsa-miR-608 and hsa-miR-638 have been reported to be involved in the occurrence, development, and progression of cancer (37, 38). Hsa-miR-4469 can act as a tumor suppressor by regulating the infiltration of inflammatory cells in colorectal cancer, and hsa-miR-4721 has been found to promote the invasion capacity of nasopharyngeal carcinoma cells (39, 40).

To determine the function of circRNA-targeted genes, GO and KEGG enrichment analyses were performed, revealing that several important signaling pathways might be involved in the detrimental effects of PEM on oocytes. In the PEM–TFI comparison groups, some target genes were enriched in the apoptosis, PI3K–AKT signaling pathway, and p53 signaling pathway, which have been reported to affect oocyte development. A systematic review of 22 articles revealed that the PI3K/AKT/PTEN pathway can regulate the progression to MII and blastulation of mammalian oocytes during *in vitro* maturation (41). Wei et al. illustrated that two downregulated long non-coding RNAs (*NEAT1* and *NORAD*) impair oocyte maturation and genome integrity by modulating PI3K–AKT pathway genes, leading to recurrent oocyte maturation arrest in women (42). The activity of the p53 tumor suppressor protein can induce cell cycle arrest or apoptosis and is related to the apoptosis of granulosa cells (43). Several experiments have confirmed the close relationship between the p53 pathway and granulosa cell death. For example, Yang et al. elucidated that the p53 pathway is involved in oxidative stress-induced apoptosis of granulosa cells using COV434, a human granulosa cell line (44). Another study reported that p53 induction in CCs impaired oocyte function and quality by disturbing maturation and fertilization in mouse models (45). In contrast, functional analysis of target genes in the PEM–OEM comparison groups demonstrated that the JAK–STAT and TGF-β signaling pathways may be involved in the different effects of PEM and OEM on oocyte quality and competence. The JAK–STAT signaling pathway is a well-established pathway involved in cell proliferation, and STAT3 signaling is extensively enriched in human primordial and primary follicles (46). Some findings have revealed the involvement of the JAK–STAT pathway in follicular development in mares and mice (47, 48). Furthermore, Frost et al. confirmed that the JAK–STAT signaling pathway contributes to the crosstalk between oocytes and CCs in humans using the COV434 cell line (49). Another enriched pathway is the TGF-β signaling pathway, which is reported to mediate the activation of primordial follicles, folliculogenesis, and oocyte–CC communication (50). In the *Caenorhabditis elegans* model, TGF-β signaling was found to regulate reproductive aging by modulating many aspects, such as oocyte morphology and quality, oocyte fertilization ability, chromosome segregation fidelity, and embryo viability and integrity (51). TGF-β is believed to be involved in the etiology of PEM. The “Warburg-like” effect induced by TGF-β, which refers to the metabolic reprogramming of glucose to lactate *via* glycolysis, may play a role in the development of PEM lesion (52). In addition, a review including 95 studies demonstrated that increased levels of TGF-β1 are related to PEM lesion development by changing ectopic endometrial and peritoneal cell metabolism and initiating neoangiogenesis, while increased levels of TGF-β ligands lead to increased attachment, invasion, and proliferation of ectopic endometrial cells (53). Thus, it is supposed that the TGF-β signaling pathway not only plays a critical role in the development of PEM lesions but may also be an important unique mechanism by which PEM damages oocyte quality and competence.

Our study had several limitations. First, the sample size for the qRT-PCR validation was small. Second, all patients in the OEM group had stage III–IV disease, while in the PEM group, all patients had stage I disease. Thus, larger samples including different stages of the OEM and PEM are warranted for further validation. Third, due to the insufficient total amount of RNA in each sample, we could not directly obtain the sequences of miRNAs. Finally, the molecular mechanisms and functions of the validated circRNAs should be investigated in the future, for example, by performing knockdown and overexpression experiments using the human granulosa cell line.

In summary, we revealed for the first time that circRNA expression patterns of CCs in patients with infertility showing different endometriosis phenotypes (OEM and PEM). We verified that the OEM and PEM groups shared 11 common circRNAs that may be involved in the common mechanisms of impaired oocyte quality in patients with OEM or PEM. circRNAs (n = 39) in the OEM group and 17 in the PEM group were identified as unique circRNAs. hsa_circ_0004872 was found to be significantly upregulated in the PEM group compared to that in the OEM and TFI groups, and downstream target genes were verified by constructing ceRNA networks and sequencing. Based on the results of functional analysis of target genes, we speculate that PEM damages oocyte competence and development *via* the PI3K–AKT and p53 signaling pathways, while JAK–STAT, especially the TGF-β signaling pathway, may be involved in a unique impairment mechanism different from that in the OEM. Our findings provide novel insights into the effects of different endometriosis phenotypes on oocytes. In addition, hsa_circ_0004872, as the PEM unique circRNA, may be used as a potential biomarker to help noninvasively identify whether infertility patients have concealed PEM and whether OEM affected patients have concomitant PEM lesions.

## Data availability statement

The datasets presented in this study can be found in online repositories. The names of the repository/repositories and accession number(s) can be found below: https://ngdc.cncb.ac.cn/gsa-human/, HRA003694.

## Ethics statement

The studies involving human participants were reviewed and approved by Institutional Review Board of the Peking Union Medical College Hospital. The patients/participants provided their written informed consent to participate in this study.

## Author contributions

XH performed sample collection and purification, analyzed the qRT-PCR data, visualized the results, and compiled the manuscript. QY contributed to the study design and edited the manuscript. All authors contributed to the article and approved the submitted version.
