# Supplementary material for: Bioinformatic analysis confirms differences in circular RNA expression profiles of cumulus cells between patients with ovarian and peritoneal endometriosis-associated infertility
Source: Front Endocrinol (Lausanne). 2023 Mar 15;14:1137235. doi: 10.3389/fendo.2023.1137235 (PMC10050890; doi:10.3389/fendo.2023.1137235)
Supplement: Supplementary file 1 [file DataSheet_1.docx]

Supplementary Material

Bioinformatic Analysis Confirms Differences in Circular RNA Expression Profiles of Cumulus Cells between Patients with Ovarian and Peritoneal Endometriosis-Associated Infertility

Xiaodi Huang, Qi Yu*

*** Correspondence:** Qi Yu: [yuqi2008001@sina.com](mailto:yuqi2008001@sina.com)

# Supplementary Figures and Tables

## Supplementary Figures


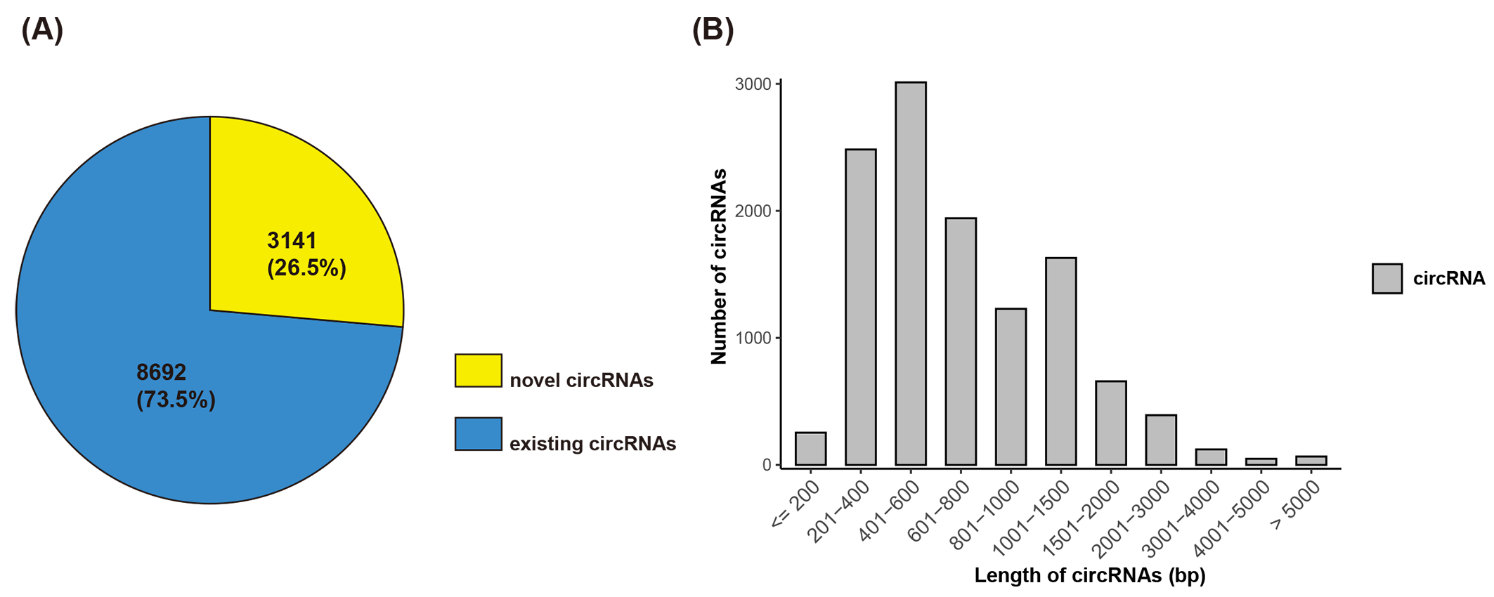


**Supplementary Figure 1.** Types and length distribution of circRNAs in CCs derived from nine samples. (A) The pie chart indicates the number and proportion of novel and existing circRNAs. (B) Histogram showing the distribution of circRNA lengths. circRNAs, circular RNAs; CCs, cumulus cells.

## Supplementary Tables

Supplementary Table 1. Primer sequences of selected circRNAs and reference genes

| Name | Forward primer sequences | Reverse primer sequences |
| --- | --- | --- |
| hsa_circ_0003638 | TGAAGGCGCTAAGGCACATA | CACAAGGAGATTCCCTGGCT |
| hsa_circ_0005205 | TGTGACCATGAGGAAATGAG | TCTTTACTTCGCCGAGATCT |
| hsa_circ_0005015 | ACACAGCCTTCAGAGCACTG | TCCAAAGAGTGTGGTTCCAA |
| hsa_circ_0138839 | GGTTTTCTGGTGCCTTTGAA | CACGAAAGACAAAGCTTCCC |
| hsa_circ_0003221 | AAATACGGCGATCATACTGG | TGGTGTGTGATTCAAGTTGG |
| hsa_circ_0004872 | TCCTGACAGAATATGTGGCC | GCAGTAGGTCTGGTGCTCAA |
| hsa_circ_0008927 | TCAGCCTGGCATTTGACAAA | TCGATGAGCTGCTCCAGTTG |
| β-actin | CATGTACGTTGCTATCCAGGC | CTCCTTAATGTCACGCACGAT |

circRNAs, circular RNAs.

Supplementary Table 2. Information on differentially expressed circRNAs in the three assemblages

| Assemblages | circRNA name | circRNA position* (+ positive-sense strand/- antisense strand) | circRNA  source genes | Length (bp) |
| --- | --- | --- | --- | --- |
| Common circRNAs in the OEM and PEM groups (n = 11) | hsa_circ_0053442 | chr2:32377587-32416163+ | *BIRC6* | 2547 |
|  | hsa_circ_0009031 | chr4:4648976-4649542+ | *STX18-AS1* | 303 |
|  | hsa_circ_0003638 | chr17:28163542-28172618+ | *NLK* | 398 |
|  | hsa_circ_0045308 | chr17:64519320-64526200+ | *CEP95* | 679 |
|  | hsa_circ_0002260 | chr5:79640850-79669028+ | *TENT2* | 743 |
|  | hsa_circ_0004425 | chr9:100498764-100516771+ | *MSANTD3-TMEFF1* | 364 |
|  | hsa_circ_0007046 | chr2:69363544-69374113- | *GFPT1* | 342 |
|  | hsa_circ_0006376 | chr8:42957093-42964474+ | *HOOK3* | 311 |
|  | hsa_circ_0005205 | chr14:61720378-61721823+ | *HIF1A* | 425 |
|  | hsa_circ_0070303 | chr4:84708908-84717016- | *WDFY3* | 463 |
|  | hsa_circ_0056992 | chr2:169946287-170029448+ | *UBR3* | 1751 |
| Unique circRNAs in the OEM group (n = 39) | hsa_circ_0000418 | chr12:69589484-69592159+ | *CCT2* | 304 |
|  | hsa_circ_0115481 | chr20:54554932-54610523+ | *DOK5* | 669 |
|  | hsa_circ_0122934 | chr3:183205875-183207823- | *MCF2L2* | 388 |
|  | hsa_circ_0019225 | chr10:94245945-94246621+ | *PLCE1* | 676 |
|  | hsa_circ_0007827 | chr11:117152440-117163892+ | *PAFAH1B2* | 418 |
|  | hsa_circ_0006251 | chr2:74046277-74048411+ | *TET3* | 2134 |
|  | hsa_circ_0002665 | chr10:5794884-5800705- | *GDI2* | 343 |
|  | hsa_circ_0006725 | chr5:95763507-95783963+ | *RHOBTB3* | 575 |
|  | hsa_circ_0042372 | chr17:19542392-19548133+ | *SLC47A1* | 320 |
|  | hsa_circ_0005015 | chr8:121628713-121629340- | *HAS2* | 627 |
|  | hsa_circ_0002484 | chr11:130260855-130261929- | *ZBTB44* | 1074 |
|  | hsa_circ_0004420 | chr2:84870224-84870785- | *TRABD2A* | 561 |
|  | hsa_circ_0017731 | chr10:12081471-12113064+ | *DHTKD1* | 2165 |
|  | hsa_circ_0000940 | chr19:45263112-45263367+ | *MARK4* | 103 |
|  | hsa_circ_0001633 | chr6:107503656-107506427+ | *SOBP* | 325 |
|  | hsa_circ_0130692 | chr6:131160058-131169273+ | *AKAP7* | 438 |
|  | hsa_circ_0042498 | chr17:27304779-27311616+ | *WSB1* | 514 |
|  | hsa_circ_0006859 | chr5:74634683-74636498- | *ENC1* | 1815 |
|  | hsa_circ_0134225 | chr7:3619079-3642105+ | *SDK1* | 415 |
|  | hsa_circ_0135763 | chr8:131940498-131970643+ | *EFR3A* | 1218 |
|  | hsa_circ_0138839 | chr9:5064882-5081861+ | *JAK2* | 1515 |
|  | hsa_circ_0004628 | chr1:41113282-41117010- | *SCMH1* | 333 |
|  | hsa_circ_0035923 | chr15:65667448-65670188- | *DENND4A* | 777 |
|  | hsa_circ_0007149 | chr4:1228198-1241519- | *CTBP1* | 495 |
|  | hsa_circ_0001246 | chr22:45700281-45740538+ | *ATXN10* | 782 |
|  | hsa_circ_0109196 | chr19:16679983-16680581+ | *TMEM38A* | 342 |
|  | hsa_circ_0002596 | chr19:2076810-2078679- | *MOB3A* | 743 |
|  | hsa_circ_0007976 | chr14:61721508-61721823+ | *HIF1A* | 231 |
|  | hsa_circ_0003856 | chr15:85113872-85115487+ | *PDE8A* | 214 |
|  | hsa_circ_0006952 | chr18:51054780-51067187+ | *AC091551.1* | 854 |
|  | hsa_circ_0002316 | chr1:58506059-58539310- | *DAB1* | 1381 |
|  | hsa_circ_0076561 | chr6:43546570-43553503- | *POLR1C* | 901 |
|  | hsa_circ_0120875 | chr2:6996915-7030215+ | *RNF144A* | 758 |
|  | hsa_circ_0107922 | chr18:12999420-13030608+ | *CEP192* | 1538 |
|  | hsa_circ_0012671 | chr1:54865302-54876047- | *DHCR24* | 633 |
|  | hsa_circ_0083196 | chr7:158735502-158739121- | *ESYT2* | 337 |
|  | hsa_circ_0002158 | chr1:8541213-8557523- | *RERE* | 308 |
|  | hsa_circ_0000118 | chr1:117402185-117420649+ | *MAN1A2* | 553 |
|  | hsa_circ_0003221 | chr8:140846259-140890769- | *PTK2* | 625 |
| Unique circRNAs in the PEM group (n = 17) | novel_circ_PGR | chr11:101041944-101126158- | *PGR* | 1009 |
|  | hsa_circ_0000153 | chr1:167765582-167776166+ | *MPZL1* | 617 |
|  | hsa_circ_0001313 | chr3:56592969-56594028+ | *CCDC66* | 468 |
|  | hsa_circ_0070421 | chr4:88475840-88479507+ | *HERC5* | 345 |
|  | hsa_circ_0025967 | chr12:45926141-45928859- | *SCAF11* | 2718 |
|  | hsa_circ_0005374 | chr1:77711746-77715868- | *USP33* | 488 |
|  | hsa_circ_0004872 | chr22:21799011-21807846- | *MAPK1* | 490 |
|  | hsa_circ_0006411 | chr5:68226289-68227009+ | *PIK3R1* | 720 |
|  | hsa_circ_0005348 | chr11:110136662-110137414+ | *RDX* | 752 |
|  | hsa_circ_0122727 | chr3:172247532-172298787+ | *FNDC3B* | 797 |
|  | hsa_circ_0018403 | chr10:50460672-50590247- | *SGMS1* | 371 |
|  | hsa_circ_0082624 | chr7:139061035-139074030- | *ZC3HAV1* | 399 |
|  | hsa_circ_0078288 | chr6:150944485-150965037+ | *MTHFD1L* | 573 |
|  | hsa_circ_0008927 | chr3:11358417-11380052+ | *ATG7* | 672 |
|  | hsa_circ_0008953 | chr2:109585125-109593119- | *SEPT10* | 383 |
|  | hsa_circ_0001367 | chr3:183643479-183651276+ | *KLHL24* | 1044 |
|  | novel­_circ_TLL2 | chr10:96405231-96413316- | *TLL2* | 344 |

* The positions of circRNAs were based on the hg 38 version. circRNAs, circular RNAs; OEM, ovarian endometriosis; PEM, peritoneal endometriosis.
